# Vitamin D level in patients with systemic lupus erythematosus: its relationship to disease course and bone mineral density

**DOI:** 10.1136/lupus-2023-000968

**Published:** 2023-08-09

**Authors:** Sergii Shevchuk, Liubov Marynych, Tetiana Malovana, Liudmyla Denyshchych

**Affiliations:** 1 Vinnytsia National Medical University, Vinnytsia, Ukraine; 2 Research Institute for Rehabilitation of Individuals with Disabilities (Educational and Scientific Treatment Facility) of Vinnytsia National Medical University, Vinnytsia, Ukraine

**Keywords:** Osteoporosis, Bone Mineral Density, Disease Activity, Glucocorticoids, Lupus Erythematosus, Systemic

## Abstract

**Objective:**

To determine vitamin D levels in patients with SLE and evaluate their relationship to bone mineral density (BMD) and the disease course.

**Methods:**

The study included 101 patients with SLE and 29 individuals in the control group. The study participants were tested for vitamin D level, erythrocyte sedimentation rate (ESR), C reactive protein (CRP), interleukin (IL)-6, osteocalcin (OC) and collagen type I C-terminal telopeptide (CTX), and the dual-energy X-ray absorptiometry was provided to assess BMD in the lumbar spine and the hip.

**Results:**

The mean serum vitamin D level was 18.98±0.88 ng/mL, and women had 25.42% lower vitamin D levels than men (p<0.05). There was no correlation between vitamin D levels and patient’s age or disease course. There was a significant inverse correlation between vitamin D levels and cumulative dose of glucocorticoids (r=−0.26) and serum inflammatory markers, particularly CRP (r=−0.39), IL-6 (r=−0.37) and ESR (r=−0.15). Vitamin D level was associated with the bone turnover markers (BTMs). In women of reproductive age with vitamin D deficiency, BMD of the lumbar spine and the hip was 9.5–23.1% higher than in those with no vitamin deficiency, respectively, and the mean lumbar spine Z-score in women of reproductive age with vitamin D insufficiency and deficiency was significantly 2.0 and 2.9 times lower than in patients with normal vitamin D level.

**Conclusions:**

Hypovitaminosis D is quite common in patients with SLE and is associated with high inflammatory activity (SLE Disease Activity Index, ESR, CRP, IL-6), severity of organ damage (Damage Index), cumulative dose of glucocorticoids, BTM changes (decrease in OC, increase in CTX) and BMD decline. Vitamin D status was not associated with the patient’s age or disease course.

**WHAT IS ALREADY KNOWN ON THIS TOPIC**

- It is known that deficiency and insufficiency of vitamin D is a widespread phenomenon among patients with SLE, which may be associated with long-term use of glucocorticoids and sunscreens, kidney damage, the presence of antibodies to vitamin D, etc. Vitamin D deficiency in patients with SLE is associated with reduced bone mineral density (BMD), while scientific information on the relationship between the level of vitamin D and bone turnover markers, as well as the duration, activity of the disease and glucocorticoid therapy, is limited.

**WHAT THIS STUDY ADDS**

- According to the results of the study, it was found that among patients with SLE, 62.3% had vitamin D deficiency, 29.7% insufficiency and 7.93% optimal level of vitamin D. Hypovitaminosis D was associated with high activity of the inflammatory process (SLE Disease Activity Index, erythrocyte sedimentation rate, C reactive protein, interleukin-6), severity of organ damage (Damage Index), cumulative dose of glucocorticoids, bone turnover marker abnormality and BMD decline. No relationship between the status of vitamin D and the age of the patients and the duration of the disease was established.

**HOW THIS STUDY MIGHT AFFECT RESEARCH, PRACTICE OR POLICY**

- The results of this study allow us to partially reveal the pathogenesis of vitamin D deficiency and, as a consequence, bone loss in patients with SLE. It is quite likely that by affecting these links of pathogenesis in the future, it is possible to prevent a decrease in the BMD in such individuals.

## Introduction

Vitamin D plays a leading role among the factors associated with autoimmune inflammatory diseases.^1^ Its deficiency is an important link in the pathogenesis of many diseases (diabetes mellitus, rheumatoid arthritis, spondyloarthritis, glomerulonephritis and hyperuricaemia). Vitamin D is known to be involved in maintaining calcium and phosphorus levels and regulating bone metabolism. Vitamin D is now increasingly recognised as a potent immunomodulator that regulates both innate and adapted immune responses.^2 3^ In recent years, interest in vitamin D has been linked to the potential for its therapeutic use to reduce inflammatory activity and improve patients’ quality of life in many autoimmune diseases. In particular, several studies have confirmed a statistically significant downward change in the SLE Disease Activity Index (SLEDAI) in patients with SLE in response to vitamin D administration,^4–8^ as well as an improvement in the total score of the Fatigue Severity Scale.^4 5^ Experimental and clinical studies have shown that vitamin D status often determines the features of the SLE clinical course, severity and activity.^9–14^

Population studies showed that vitamin D insufficiency occurs in two-thirds and deficiency in every five patients with SLE.^15–19^ Among the causes of hypovitaminosis D in SLE are kidney failure, long-term use of certain medications such as glucocorticoids (GCs), antiepileptic drugs and sunscreens, production of antibodies to vitamin D, etc.^20–23^ Vitamin D deficiency is also known to be quite common across the population of Ukraine compared with other European regions.^24^ Therefore, it is logical to determine levels of this vitamin not only in patients with SLE but in a particular, the population of this region.

In recent years, studies have shown that vitamin D deficiency in patients with SLE is strongly associated with reduced bone mineral density (BMD) determined by dual-energy X-ray absorptiometry (DEXA).^25–28^ At the same time, information on the relationship between vitamin D levels and bone turnover markers (BTMs) remains poorly elucidated today. The association of vitamin D with other risk factors for osteoporosis (age, disease duration, GC therapy) in patients with SLE also requires further investigation. The association of vitamin D with disease severity and activity is also quite controversial.^29–31^

The objective was to determine vitamin D levels in patients with SLE and evaluate their relationship to BMD and the disease course.

## Materials and methods

A total of 101 patients with SLE (90 women and 11 men) were enrolled in the study, comprising the treatment group. The study was conducted in compliance with the Key Principles of Good Clinical Practice guidelines (1996), Council of Europe Convention on Human Rights and Biomedicine (of 04 April 1997), World Medical Association Declaration of Helsinki: ethical principles for medical research involving human subjects (1964–2000) and the Order of the Ministry of Health of Ukraine No. 281 of 01 November 2000. The diagnosis of SLE was based on the American College of Rheumatology (ACR) criteria (2019) and formulated according to the classification recommended by the Association of Rheumatologists of Ukraine (2002).^32^ The disease activity in the group of patients with SLE was determined using the SLEDAI score. The degree of internal organ damage was assessed according to Systemic Lupus International Collaborating Clinics/ACR Damage Index (DI).^33^

The control group included 29 age-matched and sex-matched subjects who showed no evidence of musculoskeletal system damage and no evidence to suggest rheumatological pathology. The patients’ mean age in the treatment group was 53.45±2.64 years (from 23 to 65 years), and that of the control group was 56.79±2.30 years (ranging from 22 to 66 years).

The vitamin D level was determined using a 25-ОН Vitamin D Total (Vit-D Direct) Test System (Monoblind, USA). Blood samples from all patients were taken in the summer period (from May to October). Patients who used sunscreens and received additional vitamin D supplements were not included in this study. According to laboratory standards, the vitamin D status was characterised as optimal (30–50 ng/mL), insufficient (20–30 ng/mL) and deficient (<20 ng/mL).

In all patients, the cumulative dose of GCs was calculated as the product of the daily GC dose by the number of days of drug administration throughout the SLE treatment period, expressed as the methylprednisolone equivalent.

The serum C reactive protein (CRP) level was determined by immunoenzyme method using a standard kit, manufactured by Diagnostic Automation, USA. The serum proinflammatory cytokine interleukin (IL)-6 level was determined by immunoenzyme technique using a standard kit manufactured by Calbiotech, Germany.

The serum osteocalcin (OC) was determined by immunoenzyme method using the commercial N-MID Osteocalcin ELISA kit (Immunodiagnostic Systems Nordic, Denmark).

The collagen type I C-terminal telopeptide (CTX) concentration was determined by immunoenzyme technique using the commercial MicroVue CICP EIAKit kits (Quidel, USA).

The BMD changes in persons of both groups at the lumbar spine (L1–L4) and the hip (femoral neck and total hip) were determined by the DEXA on a Hologic Discovery Wi (S/N 87227) apparatus. The results of the BMD measurements were expressed in absolute values, T-score and Z-score. The BMD test measures the bone mineral content per unit area scanned (g/cm^2^).

Osteoporosis was diagnosed in postmenopausal women if the T-score at the lumbar (L1–L4) spine or the hip (femoral neck and total hip) was −2.5 SD or less. In women of reproductive age, the Z-score was used to determine BMD. A Z-score value ≤−2.0 SD was categorised as below the expected range for age.

Statistical processing was made using SPSS Statistics software, Version 26. Multiple comparisons for unconjugated samples were performed using Kruskal-Wallis H test with the Bonferroni correction. The significance of differences between two unconjugated samples was compared using the Student’s t-test. Differences at p<0.05 were considered significant.

## Results

The vitamin D level analysis in patients with SLE (table 1) showed that its mean serum value was 18.98±0.88 ng/mL, and this indicator was statistically significantly lower in women than in men by 25.42% (p<0.05). Thus, among women with SLE, 59 (65.6%) patients had vitamin D deficiency, 25 (27.8%) registered vitamin D insufficiency and only 6 (6.7%) had optimal levels. Among men, four (36.4%) patients had vitamin D deficiency, five (45.5%) patients registered insufficient levels and two (7.9%) had normal levels. In the control group, the normal 25-hydroxy-cholecalciferol (25(OH)D) levels were detected in 10 (34.5%) men, vitamin D insufficiency in 10 (34.5%) patients and in 9 (31%) registered deficiency.

**Table 1:** Vitamin D levels in patients with SLE

Parameter	M±m	Vitamin D, n (%)			P
		Optimal level (30–100 ng/mL)	Insufficiency (20–29 ng/mL)	Deficiency (<20 ng/mL)	
		1	2	3	
**Women with SLE**					
n (%)	90	6 (6.67)	25 (27.78)	59 (65.56)	
Vitamin D, ng/mL	18.30±0.92	40.27±3.20	24.32±0.52*	13.52±0.59*	0.0001
**Men with SLE**					
n (%)	11	2 (18.18)	5 (45.45)	4 (36.36)	
Vitamin D, ng/mL	24.55±2.33†	35.5±2.00	26.60±1.12†	16.50±1.04*†	0.013
**All patients with SLE**					
n (%)	101	8 (7.93)	30 (29.7)	63 (62.3)	
Vitamin D, ng/mL	18.98±0.88	39.08±2.65	24.70±0.49*	13.72±0.55*	0.0001
**Control group**					
n (%)	29	10 (34.5)	10 (34.5)	9 (31.0)	
Vitamin D, ng/mL	27.2±1.3	34.39±0.88	27.24±0.45*	17.75±0.60*	0.026

P=the probability of differences between groups 1, 2, 3 by Kruskal-Wallis H test with the Bonferroni correction.

*The probability of differences compared with the value in the optimal level group (p<0.05).

†The probability of differences (p<0.05) between men and women in the respective groups.

The study found no relationship between vitamin D levels and patient age (table 2). The proportion of individuals with vitamin D deficiency in the young, middle-aged and elderly patient groups was almost comparable, ranging from 62% to 67%. As for the mean vitamin D values, they were the lowest in the elderly.

**Table 2:** Relationship between vitamin D level and age in patients with SLE

No	Age group, years old	M±m	Vitamin D, n (%)		
			Optimal level (30–100 ng/mL), n=8	Insufficiency (20–29 ng/mL), n=30	Deficiency (<20 ng/mL), n=63
1	Young aged up to 44 (n=45)	19.32±1.48	4 (8.9)	13 (28.9)	28 (62.2)
2	Middle-aged 45–59 (n=53)	18.87±1.11	4 (7.5)	16 (30.2)	33 (62.3)
3	Elderly aged 60 and over (n=3)	15.97±2.80	0	1 (33.3)	2 (66.7)

The study did not reveal the relationship between disease duration and vitamin D level in patients with SLE (table 3). So, vitamin D deficiency was found in 47.8% of patients with disease duration of 5–10 years, in 88.2% of individuals with up to 5-year disease duration and in 63.3% of patients with >10-year history of disease. The mean vitamin D values did not differ significantly between groups with different types of disease duration.

**Table 3:** Relationship between vitamin D levels, disease duration and cumulative dose of GCs in patients with SLE

Parameter	M±m	Vitamin D, n (%)		
		Optimal level (30–100 ng/mL), n=8	Insufficiency (20–29 ng/mL), n=30	Deficiency (<20 ng/mL), n=63
Relationship to disease duration				
<5 years (n=17)	16.08±1.34	1 (5.8)	2 (11.8)	14 (88.2)
5–10 years (n=23)	20.28±2.28	1 (4.3)	11 (47.8)	11 (47.8)
>10 years (n=61)	19.30±1.04	6 (9.83)	17 (27.8)	38 (63.3)
Correlation coefficient		0.05		
Relationship with cumulative dose of GCs				
Cumulative dose of GCs <42.8 g (n=50)	21.63±1.43	7 (14.0)	17 (34.0)	26 (52.0)
Cumulative dose of GCs >42.8 g (n=51)	16.39±0.90*	1 (1.9)	13 (25.5)	37 (72.5)
Correlation coefficient		−0.26*		

*The probability of differences in relation to the group with a cumulative dose of GCs of <42.8 g (p<0.05).

GCs, glucocorticoids.

The study revealed a negative association between cumulative dose of GCs and vitamin D level. In particular, its low concentration was associated with a high GC dose. Thus, with a cumulative dose of GCs of >42.8 g, the mean vitamin D level was 31.7% lower than in the group with a cumulative dose of GCs of <42.8 g. In the high-dose GC group, the proportion of individuals with vitamin D deficiency was 72.5%, while in the low-dose GC group, it was 52%. The results of the correlation analysis revealed a statistically significant inverse correlation (r=−0.26) between the cumulative dose of GCs and vitamin D level.

There was a similar relationship between the inflammatory marker concentration, the severity of organ damage and serum vitamin D status (table 4). In patients with the optimal vitamin D levels (30–100 ng/mL), CRP, IL-6 and erythrocyte sedimentation rate (ESR) were 8.21±0.57, 12.08±0.55 pg/mL and 18.29±4.21 mm/hour, respectively; in other words, they were 19.4%, 27.2% and 52% lower than in patients with vitamin D insufficiency (p<0.05). After the evaluation of the mean values in the vitamin D-deficient group, the differences were detected even greater. In particular, the mean values of the laboratory markers of inflammation in this group were 42.7%, 44.2% and 43% higher than in the group of patients with optimal vitamin D levels. Similar patterns were found when assessing the relationship between the total activity index of SLEDAI and DI, which was 62–79% higher in the group of patients with vitamin D deficiency than in the group with an optimal vitamin D level (p<0.05). Correlation analysis confirmed a significant relationship between the level of inflammatory markers and serum vitamin D level in patients with SLE. It should be noted that vitamin D deficiency was more strongly correlated with increased IL-6 (r=−0.37) and CRP (r=−0.39), as well as with DI (r=−0.36).

**Table 4:** Relationship between inflammatory activity (ESR, CRP, IL-6, SLEDAI, DI) indicators and serum vitamin D levels, M±m

Parameter	Vitamin D level			r
	Optimal level (30–100 ng/mL)	Insufficiency (20–29 ng/mL)	Deficiency (<20 ng/mL)	
	n=8	n=30	n=63	
CRP	8.21±0.57	9.81±0.61	11.72±0.62*	−0.39†
ESR, mm/hour	18.29±4.21	27.73±2.85	26.13±1.84	−0.15
IL-6, pg/mL	12.08±0.55	15.57±0.91*	17.43±0.59*	−0.37†
SLEDAI, points	10.13±0.85	15.20±1.28*	16.46±0.76*	−0.26†
DI, points	2.25±0.16	3.47±0.33*	4.03±0.26*	−0.36†

*Indicates significant differences relative to patients with optimal vitamin D levels.

†Indicates the significant correlation coefficient values.

CRP, C reactive protein; DI, Damage Index; ESR, erythrocyte sedimentation rate; IL-6, interleukin-6; SLEDAI, SLE Disease Activity Index.

The vitamin D level was associated with the BTMs, as indicated by the proportional change in the synthesis (OC) and resorption (CTX) marker concentrations related to the decreased vitamin D levels (table 5). In particular, the mean levels of OC were 14.9% lower, and the mean levels of CTX were 36% higher in vitamin D-deficient patients than in those with optimal vitamin D levels. The results of correlation analysis confirmed the strong relationship between vitamin D and OC levels (r=0.30) and CTX (r=−0.26).

**Table 5:** Relationship between bone turnover markers (osteocalcin, CTX) and serum vitamin D levels in patients with SLE, M±m

Parameter	No	Bone turnover markers	
		Osteocalcin, ng/mL	CTX, ng/mL
Optimal vitamin D level (30–100 ng/mL)	1	15.40±1.26	0.94±0.10
Vitamin D insufficiency (20–29 ng/mL)	2	15.13±0.78	1.21±0.07*
Vitamin D deficiency (<20 ng/mL)	3	13.41±0.41	1.28±0.04*
Correlation coefficient	4	0.30†	−0.26†

*Indicates significant differences relative to patients with optimal vitamin D levels.

†Indicates significant correlation coefficient values.

CTX, collagen type I C-terminal telopeptide.

In the next part of our study, we have analysed the relationship between vitamin D levels and BMD in patients with SLE. Given the small proportion of men examined, the table represents data for women only. Vitamin D levels were associated with the impaired bone state, determined by Z-score and T-score, depending on reproductive age and BMD (table 6). Thus, in the group of female patients of reproductive age with vitamin D deficiency, the mean Z-score value was 2.9 times (for lumbar spine) and 5.9 times (for hip) lower than in the group of patients with no hypovitaminosis D, while BMD was 9.5–23.1% higher, respectively. Similar differences were found in the postmenopausal women. Thus, the mean T-score value for lumbar spine in women with vitamin D deficiency was 1.6 times lower compared with this indicator in women with optimal vitamin D level, while the mean T-score value for hip differed by 1.26 times for similar female patient groups. BMD in women with vitamin D deficiency was 3.4–11.1% higher than in those without hypovitaminosis D, although these values did not reach statistically significant values.

**Table 6:** Relationship between bone mineral density (BMD) in women of different reproductive ages with SLE and different vitamin D levels (M±m)

Parameter		Characteristics	Optimal level (30–100 ng/mL)	Insufficiency (20–29 ng/mL)	Deficiency (<20 ng/mL)
Women in the reproductive period			n=5	n=14	n=33
1	Z-score ≤−2.0 SD	Lumbar spine	0 (0%)	1 (7.14%)	7 (21.2%)
		Hip	0 (0%)	1 (7.14%)	26 (78.8%)
2	Z-score >−2.0 SD	Lumbar spine	5 (100%)	13 (92.9%)	29 (87.9%)
		Hip	5 (100%)	13 (92.9%)	4 (12.1%)
3	Z-score, M±m, SD	Lumbar spine	−0.38±0.36	−0.77±0.21*	−1.09±0.13*
		Hip	−0.16±0.17	−0.26±0.24	−0.95±0.15
4	BMD, g/cm^2^	Lumbar spine	0.78±0.03	0.91±0.03	0.96±0.01
		Hip	0.84±0.03	0.91±0.03	0.92±0.03
Postmenopausal women			n=1	n=11	n=26
5	Osteoporosis, T-score −2.5 SD and less	Lumbar spine	0 (0%)	2 (18.2%)	9 (34.6%)
		Hip	0 (0%)	0 (0%)	6 (23.1%)
6	Osteopenia, T-score from −1.0 SD to −2.5 SD	Lumbar spine	1 (100%)	7 (63.6%)	13 (50.0%)
		Hip	1 (100%)	7 (63.6%)	13 (50.0%)
7	Normal, T-score from +2.5 SD to −1.0 SD	Lumbar spine	0 (0%)	2 (18.2%)	4 (15.4%)
		Hip	0 (0%)	4 (36.4%)	7 (26.9%)
8	T-score, mean value, SD	Lumbar spine	−1.22±0.00	−1.57±0.46*	−1.95±0.18*
		Hip	−1.20±0.00	−1.09±0.22	−1.51±0.21*
9	BMD, g/cm^2^	Lumbar spine	0.81±0.00	0.79±0.05	0.90±0.03
		Hip	0.87±0.00	0.85±0.03	0.90±0.04

*Indicates a significant difference in patients with optimal vitamin D levels (p<0.05).

## Discussion

The study found that 93 (92.07%) patients with SLE had insufficient or deficient vitamin D levels, and only 8 (7.93%) had normal levels. Numerous studies have reported a high incidence of vitamin D deficiency in patients with SLE.^34–36^ In particular, according to the North American study, 66.7% of patients with SLE had the 25(OH)D concentration of less than 80 nmol/L (32 ng/mL), and 17.9% had less than 40 nmol/L (16 ng/mL).^34^ Similar results were obtained in the study conducted in Saudi Arabia, where 98.8% of patients with SLE had suboptimum vitamin D levels or vitamin D deficiency.^37^ In contrast, according to Bultink *et al*, vitamin D deficiency was found in only 8% of 107 patients.^38^ These contradicting results may be due to differences in study design, patient group characteristics, 25(OH)D reference values, etc.

We found that the mean 25(OH)D level was 18.98±0.88 ng/mL, and women had statistically significantly lower vitamin D levels than men by 25.42% (p<0.05). There was no evidence of gender differences in vitamin D status found in the literature available.

Although the lowest 25(OH)D level was found in the group with the shortest disease duration, in general, the levels of the vitamin tested had no associative relation to disease duration. The literature data also show no correlation between disease duration and vitamin D levels.^16 34 39–41^ Instead, Adel *et al* argue that low vitamin D levels (insufficiency or deficiency) were mostly observed in patients with a long history of SLE.^42^ Obviously, there are other (internal organ damage, disease activity, pharmacotherapy administration, genetic polymorphism of the receptor) factors that determine the vitamin D deficiency in patients with SLE. Regarding the genetic polymorphism of the receptor, its presence can considerably alter vitamin D potency and create vitamin deficiency even in the case of adequate vitamin D intake.^43^ By various estimates, the contribution of genotype to the serum vitamin D level variations ranges from 23–43% to 77–80%.^44 45^

It is known from the literature that long-term GC therapy affects vitamin D metabolism. Animal studies have shown that a high GC dose stimulates 24-hydroxylase activity and moderately reduces 1-alpha-hydroxylase activity in the kidneys.^22 46–48^ Functional cooperation between the GC receptor, the transcription factor C/EBPβ and the vitamin D receptor has been observed that increases 24-hydroxylase transcription.^49^ Thus, the experimental data allow to formulate the concept that vitamin D deficiency develops with long-term GC therapy due to accelerated 25(OH)D catabolism. However, some studies have found both reduced and normal 25(OH)D levels in patients who were administered GCs for a long time.^50 51^ We found strong associative inverse correlation (r=−0.26) of cumulative dose of GCs with vitamin D levels. Thus, in the group of patients with a cumulative dose of GCs of >42.8 g, the mean vitamin D level was 31.7% lower than in the patient group with <42.8 g. In the high-dose GC group, the proportion of individuals with vitamin D deficiency was 72.5%, while in the low-dose GC group, it was 52%.

Another pathogenetic factor adversely affecting vitamin D levels in patients with SLE is the systemic inflammatory process. CRP is considered a standard marker of the inflammatory process, and its synthesis is regulated by IL-6.^52^ As is known, CRP increases in persons with an active course of SLE.^53 54^ Thus, according to the literature, in patients with SLE, an increase in CRP is associated with active serositis, arthritis or myositis.^55^ Thus, the negative association of vitamin D content with an increase in CRP may be an additional indication of the influence of an active inflammatory process. In particular, SLEDAI and internal organ DI were statistically significantly higher (by 62.3% and 79%, respectively) in the vitamin D-deficient group compared with the patients with optimal vitamin levels. The decreased 25(OH)D levels in patients with SLE were associated with increased levels of ESR, proinflammatory mediators CRP and IL-6. Thus, in individuals with 25(OH)D deficiency, the levels of ESR, CRP and IL-6 were on average 42–44% higher than in the case of optimal 25(OH)D levels. The correlation analysis results revealed further evidence that the decreased 25(OH)D level is associated with inflammatory activity, namely with marked cytokinaemia (IL-6 (r=−0.37) and CRP (r=−0.39)). Numerous studies have reported associations between vitamin D concentrations and SLE severity. For example, Shahin *et al,* investigating vitamin D status and its relationship to clinical and laboratory markers of SLE activity in 57 patients, found that hypovitaminosis D induced ANA production and is associated with high serum IL-23 and IL-17 levels, ultimately contributing to increased inflammatory activity.^19^ An inverse proportional relationship between vitamin D levels and SLE activity measured by the SLEDAI was confirmed in the study conducted by Yao *et al*, including 68 females of reproductive age and 12 males with SLE.^56^ The same conclusion was made by other researchers.^57–59^ In contrast, Ruiz-Irastorza *et al*, as well as Miskovic *et al*, found no association between vitamin D insufficiency or deficiency and the SLEDAI in patients with SLE.^16 60^

In recent years, more and more evidence has emerged regarding the immunoregulatory effects of vitamin D. The effect of vitamin D on the specific immune response occurs by suppressing T cell proliferation and switching T helper phenotypes, reducing the immune response of type 1 T helpers, inhibiting the production of type 1 proinflammatory cytokines such as IL-12, interferon-γ, IL-6, IL-8, tumour necrosis factor (TNF)-α and IL-9. At the same time, vitamin D enhances the production of type 2 anti-inflammatory cytokines, such as IL-4, IL-5 and IL-10. This regulation of cytokines is mainly mediated by blocking the activation of nuclear factor kappa B (NF-κB) p65 through the enhancement of the NF-kB inhibitory protein IkBa. In addition, calcitriol stimulates regulatory T cells and natural killer cells, and enhances apoptosis induced by dendritic cells and T lymphocytes.^61–63^ Vitamin D also induces a shift in macrophage polarisation from the proinflammatory M1 phenotype to the anti-inflammatory M2 phenotype, inhibits dendritic cell differentiation, decreases the expression of major histocompatibility complex (MHC) class II molecules, costimulatory molecules and CD54 (adhesion molecules) on their surface, reduces production and increases IL-6 synthesis. Inhibition of dendritic cell differentiation and maturation prevents autoimmunity and promotes immunological tolerance. This is explained by the fact that antigen presentation by mature dendritic cells induces an immune response, whereas antigen presentation by immature dendritic cells induces tolerance.^61 64 65^ Calcitriol inhibits the proliferation of B cells, their differentiation into plasma and memory B cells, and promotes apoptosis.^66^ Considering that excessive autoantibody synthesis by B lymphocytes is a key element in pathogenesis of many autoimmune diseases, in particular SLE,^67 68^ it can be concluded that vitamin D influence on B cells causes inhibition of autoantibody synthesis. Thus, it is obvious that vitamin D can regulate at least some immune reactions, and its role is predominantly anti-inflammatory, which helps to prevent hyperinflammation and autoimmunity.

In the next part of the work, we analysed the relationship between vitamin D status and levels of bone resorption (CTX) and synthesis (OC) marker levels and BMD assessed by DEXA. The mean synthesis marker levels were found to decrease by 14.9% in vitamin D deficiency and resorption marker levels to increase by 36% compared with the group with no hypovitaminosis. As for the structural changes in bone tissue, in women of reproductive age, the most changes in BMD were observed in the group with hypovitaminosis D. Thus, BMD of the lumbar spine and hip was 9.5–23.1% higher in subjects with vitamin D deficiency, respectively, than in women with normal vitamin D level. At the same time, the mean Z-score value of the lumbar spine in women of reproductive age with vitamin D insufficiency and deficiency was statistically significantly 2.0 and 2.9 times lower than in patients with normal ranges of vitamin D. In postmenopausal women with SLE, an overwhelming number of individuals who showed evidence of osteoporosis (measured by the T-score) of both the lumbar spine and the hip were vitamin D deficient. The mean BMD of both areas was lower in postmenopausal women with vitamin D insufficiency and higher in those with optimal vitamin D levels, although the differences did not reach statistically significant values. Concerning the literature data, the Chinese study involving 60 patients with SLE revealed a positive relationship between vitamin D levels and OC, C3, C4 and BMD of the L1–L4 vertebrae and femoral neck.^69^ The recent study conducted by Tianle *et al* enrolling 391 females with SLE demonstrated a significant reduction in markers of bone formation and an increase in markers of bone resorption in patients with SLE and vitamin D deficiency.^70^ The OC levels correlated negatively with the SLEDAI and positively with CTX (ratio of the measured CTX value to the age/sex-appropriate upper normal limit) in the study conducted by Sarkissian *et al*.^71^ In contrast to the findings of Dhillon *et al*, who concluded that OC level decrease in patients with SLE is associated with GC therapy,^72^ recent publications show decreased OC levels in patients with SLE at the time of diagnosis with no history of GC therapy.^69 73 74^ It is likely that bone mass loss in SLE results from an increase in osteoclast activity and a decrease in osteoblast activity, which occurs naturally in chronic inflammation. Activation of the receptor for NF-κB (RANKL) ligand and TNF increases osteoclast maturation and activity. IL-1 and IL-6 appear to play a similar role in bone metabolism.^75^ Low-density lipoproteins, which may be elevated in SLE, also contribute to T lymphocyte activation, RANKL and TNF induction, and osteoblast quantity reduction.^76^ However, all mechanisms of a relationship between SLE activity, vitamin D levels, BMD, and markers of bone synthesis and resorption are not precisely known and require further investigation.

Our study has several limitations. First, it was conducted with only one measurement of serum 25(OH)D levels, and second, it included mainly patients with high inflammatory activity. The advantages of the study are its multifactorial nature, that is, the role of disease course factors, inflammatory process activity in the vitamin D status formation, and the relationship with the BTMs and changes in BMD according to DEXA.

## Conclusions

To summarise the results, we can acknowledge that hypovitaminosis D is quite common in patients with SLE. Only 7.93% of patients with SLE had optimal vitamin D levels, 29.7% of patients had a 25(OH)D insufficiency and 62.3% of patients with SLE had a 25(OH)D deficiency. Hypovitaminosis D was associated with high inflammatory activity (SLEDAI, ESR, CRP, IL-6), severity of organ damage (DI), cumulative dose of GCs, BTMs (OC, CTX) and BMD. Vitamin D status was not associated with the patient’s age or disease duration.

## Data Availability

All data relevant to the study are included in the article or uploaded as supplemental information.
